# The brain structure and function alterations in tension-type headache: A protocol for systematic review and meta analysis

**DOI:** 10.1097/MD.0000000000020411

**Published:** 2020-06-12

**Authors:** Jun Zhou, Shirui Cheng, Han Yang, Lei Lan, Yijia Chen, Guixing Xu, Zihan Yin, Zhengjie Li, Mailan Liu

**Affiliations:** a The Acupuncture and Tuina College, the 3rd Teaching Hospital, Chengdu University of Traditional Chinese Medicine, Chengdu, Sichuan; b College of Acupuncture & Moxibustion and Tuina, Hunan University of Chinese Medicine, Hunan; c The School of Basic Medicine of Air Force Medical University, Xi’an; d Acupuncture-Brain Research Center, Chengdu University of Traditional Chinese Medicine, Chengdu, Sichuan, China.

**Keywords:** meta-analysis, neuroimaging, protocol, systematic review, tension-type headache

## Abstract

**Objective:**

The aim of this systematic review and meta-analysis is to improve the understanding of the pathophysiology of tension-type headache (TTH), as well as propose avenues for future neuroimaging studies of TTH.

**Methods:**

From the inception dates to May 1, 2020, a systematic literature will search in Medline (Ovid SP), Embase (Ovid SP), Cochrane Central Register of Controlled Trials, Web of Science, and 4 Chinese databases without limitation on language or publication. Additionally, International Clinical Trials Registry Platform , reference lists, and relevant gray literatures will be searched. After screening of eligible references, included studies will be determined according to included criteria, and then data extraction and a methodological quality assessment with a customized checklist will be conducted. Each process will be independently implemented by 2 reviewers, any disagreement will be resolved by consensus to the third researcher. If the extracted data is feasible, anisotropic effect-size version of signed differential mapping will be conducted to perform the meta-analysis of the structural and functional brain alterations in TTH patients.

## 1 Introduction

Tension-type headache (TTH) is the most common primary headache worldwide, characterized by typically bilateral, pressing, or tightening in quality, of mild-to-moderate intensity, and not aggravated by daily activities.^[1,2]^ According to the Global Burden of Diseases, Injuries, and Risk Factors Study 2016, TTH ranks the third in terms of global prevalence (30%–78%),^[2]^ as well as the sixth in terms of global incidence, both being higher than any other type of headache.^[3]^ TTH, a major health problem, usually leads to symptoms like temporomandibular joint disorders,^[4,5]^ fibromyalgia^[6,7]^ and sleep disturbances,^[8]^ anxiety, and depression,^[9,10]^ which brings substantial economic burdens to the public and society.^[1,11,12]^ However, the treatment options for TTH are not satisfactory, which may result from the incomplete understanding of TTH pathophysiology.

Previous studies on pathophysiology believed that the origin of headache in TTH patients could be attributed to excessive muscle contraction, ischemia, and inflammation of head and neck muscles. However, the opinion of peripheral origin of headache in TTH was challenged and questioned by some studies.^[13–15]^ One opinion that peripheral muscle pathology was a cause of TTH still lacking support of strong evidence. Recent decades, researchers found that chronic TTH patients showed decreased tolerance thresholds to mechanical, thermal, and electrical stimuli.^[16,17]^ It has also been demonstrated that chronic TTH patients had a qualitatively alteration of pain perception and generalized hyperalgesia.^[18,19]^ Based on those studies, it has been suggested that occurrence of chronic TTH may be due to central sensitization of neurons at spinal dorsal horn, trigeminal nucleus, or supraspinal neurons, and decreased descending inhibition from supraspinal structures induced by continuous nociceptive input from pericranial muscles and myofascial tissues.^[20]^

Neuroimaging has led to advances in the realization of TTH pathophysiology in vivo, especially at the level of supraspinal structures. The neuroimaging studies reported that TTH patients had abnormal brain function,^[21,22]^ altered brain gray matter intensity, and volume,^[23–25]^ and impaired white matter tracking.^[26,27]^ However, not all the studies reported entirely consistent findings, which may result from sample size, demographics and clinical characteristics of the patients, magnetic resonance imaging (MRI) modalities, as well as image acquisition techniques and analysis. Besides, there was no integrated study summarizing the scattered evidence of individual studies, and the central sensitization and impaired descending inhibition associated with TTH remains unclear. Therefore, it is necessary to carry out a comprehensive and holistic systematic review and meta-analysis to integrate the existing neuroimaging studies to improve the knowledge of the central factors contributing to TTH.

In this study, anisotropic effect-size version of signed differential mapping (AES-SDM) (http://www.sdmproject.com)^[28]^ will be used to perform the meta-analysis of the structure and function brain alterations in TTH patients in order to answer the following questions.

1) Are there concordant functional or structural changes in the foci (increase or decrease) in TTH?
2) Are these functional and structural changes concurrent with each other?
3) What can be deduced from functional and structural changes in TTH?

We will describe how these studies have helped our realization of the pathophysiology of TTH, discuss their limitations, and propose avenues for future research using MRI to study TTH.

## 2 Methods and design

### 2.1 Study registration

This protocol was conducted in accordance with the Preferred Reporting Items for Systematic Review and Meta-Analysis Protocols 2015 statement.^[29]^ The planned review strategy was registered on PROSPERO (www.crd.york.ac.uk) (CRD42020153320).

### 2.2 Eligibility criteria

In order to provide a thorough, best practice analysis of aberrant activation in patients with TTH, we will apply well-defined inclusion and exclusion criteria below.

#### 2.2.1 Type of study

Randomized controlled trials and observational studies including cohort and case-control capable of extracting original neuroimaging data will be included.

#### 2.2.2 Study design

Neuroimaging studies focused on differences in brain structure and function alterations between TTH patients and healthy controls (HC). If baseline neuroimaging data are reported, longitudinal studies will be considered. Structural MRI and resting-state functional MRI will be included. Any data obtained from the same participants using multimodal neuroimaging techniques will be collected separately in this study.

#### 2.2.3 Type of participant

Studies involving adult TTH patients and parallel HC will be considered for inclusion.

#### 2.2.4 Exposure

The diagnosis criteria for Patients with TTH should be based on the diagnostic criteria of the International Classification of Headache Disorders (Headache classification committee of the International Headache Society) version 1 to 3 from 1988 to 2018.^[3,30,31]^ Patients with migraine or with other types of headaches will be excluded. Some studies enrolling patients without clear discrimination of TTH diagnosis criteria will be considered after comprehensive full-text assessment or contacting with the authors.

#### 2.2.5 Type of comparators

Participation of a parallel healthy control group in the study will be required for inclusion in the current review. HC must have no prior diagnosis of TTH at enrolment, and this must be verified by clinical examination during the study. Studies will be excluded if the healthy control group was lacking.

#### 2.2.6 Outcome measurements

The structure and function alterations of brain in patients with TTH will be set as the primary outcomes. The outcomes related to structural alterations are grey matter density or volume. The outcomes related to functional alterations are as following: brain functional activity (fMRI based on blood-oxygen-level-dependent signal or cerebral blood flow), brain molecular metabolism (positron emission tomography or single-photon emission CT). Clinical variables will be set as the secondary outcomes, such as the headache frequency, headache intensity and duration of headache, symptom-related scales (such as Visual Analogue Scale and Headache Impact Test-6), quality of life (QoL) questionnaire (WHO-QoL Questionnaire-Brief version, the scores of Short Form-36 subscales), and emotional scales (Hamilton Depression Scale, Hamilton Anxiety Scale).

### 2.3 Search strategy

#### 2.3.1 Primary sources

Based on the principle of combining free words with subject words, the following 8 electronic databases from inception to May 2020 will be searched: 4 English electronic databases (Medline (Ovid SP), Embase (Ovid SP), Web of Science, and CENTRAL) as well as 4 Chinese electronic databases (Chinese National Knowledge Infrastructure, China Biology Medicine disc, Chinese Scientific Journals Database, and Wan-fang data). The literature search will be constructed around search terms for TTH, neuroimaging and search terms for various imaging techniques, and adapted for each database as necessary. Taking MEDLINE (English) and China Biology Medicine (Chinese) as examples, the specific search strategies are shown in Table 1.

**Table 1:**
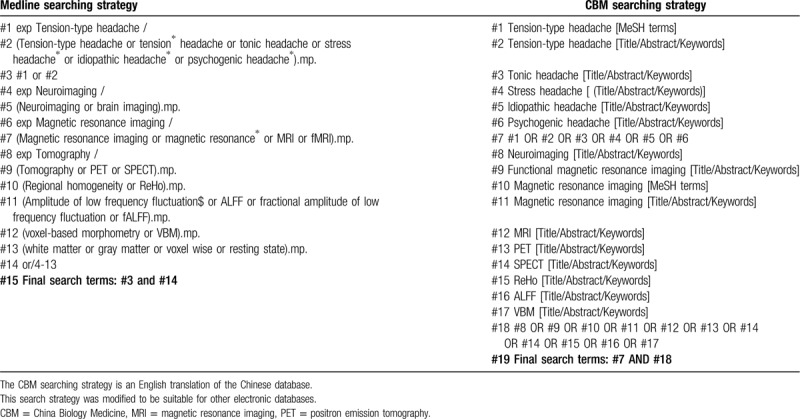
Searching items for identifying articles in Medline (English) and China Biology Medicine (Chinese).

#### 2.3.2 Supplementary sources

Additional separate searches will be run plus additional sources International Clinical Trials Registry Platform and the snowballing strategy will be employed to find other possible eligible studies from the reference lists of the articles identified for inclusion. Moreover, the Health Management Information Database, the National Technical Information Service, and OpenSIGLE Database will be searched for relevant grey literatures.

### 2.4 Data collection

#### 2.4.1 Studies selection

**Figure 1:**
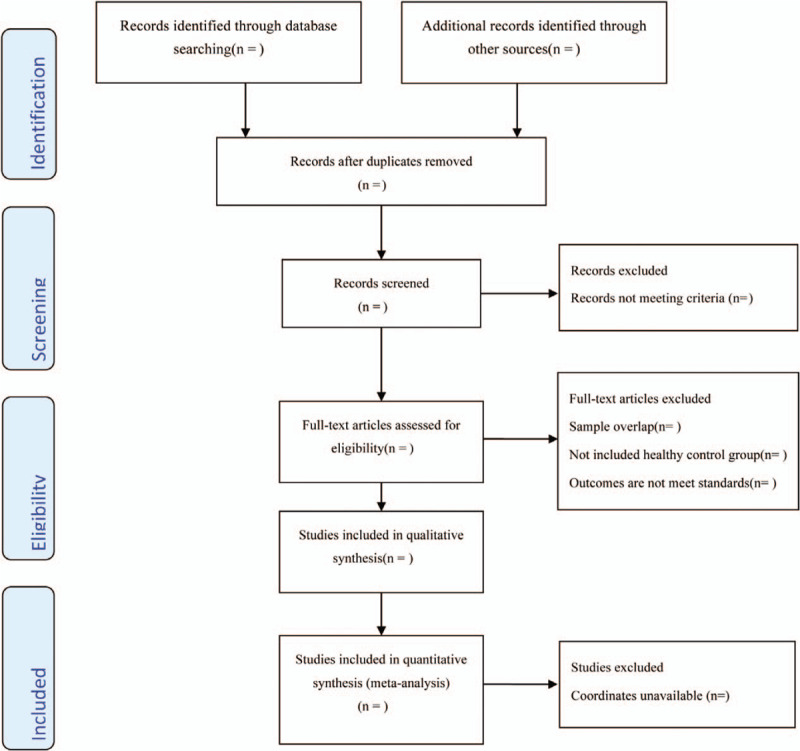
Flow diagram of study selection process.

The retrieved literatures will be imported in Endnote software 9.1 to be managed and to remove duplicate. Based on the pre-established inclusion and exclusion criteria, 2 researchers (JZ and HY) will screen through the titles and abstracts independently, then the full text will be screened for the second selection. The cross-checking will be made for the included studies. The Preferred Reporting Items for Systematic Reviews and Meta-Analyses flow diagram^[32]^ will be used to report the process of the selection. Each reason for exclusion of any literature will be listed in details (Fig. 1). If there is a disagreement, the third researcher (ZJL) will come forward to resolve.

### 2.5 Data extraction

Two researchers (ZHY and LL) will extract data into a pre-designed form independently, including information of publication (title, country, or region, publishing year, first author, and fund support), methodology details (demographic characteristics of participants, diagnostic criteria, sample size, imaging modalities, data analysis strategies, TTH-related cognitive-behavioral models, and clinical variables), results (the alterations of cerebral regions, the value of clinical characteristics, and the correlations between imaging data and clinical data). Besides, the coordinates, inference/acquisition space, z-statistics space or equivalents will be extracted. Corresponding author will be contacted for any insufficient or missing data. If no accurate data being provided after 8 weeks, the study will also be included and marked with insufficient or missing information. If there is a disagreement, the third researcher (ZJL) will come forward to resolve.

### 2.6 Dealing with missing data

For insufficient or missing primary data, the related corresponding authors will be contacted for the information. If the missing data cannot be obtained, the studies will only be included for narrative analysis.

### 2.7 Quality assessment

At present, there is no consensus about the standard for quality assessment of neuroimaging studies. Previous neuroimaging systematic reviews usually set the quality assessment tools based on their own studies.^[33–36]^ In this study, we will set a suitable checklist which including 11 items (Table 2) to assess the quality of the included studies after referring to the similar lists used in previous meta-analytic studies.^[37,38]^ We will mark each item as 1 (Yes) or 0 (No or Unclear), and the summation of items generates an overall quality score (0–11 points). The quality of each study will be defined as high (9–11 points), medium (5–8 points), or low (0–4 points). Please check Table 2 for details. Two researchers (JZ and SRC) will assess the quality independently based on the checklist. If there is a disagreement, the third researcher (ZJL) will come forward to resolve.

**Table 2:**
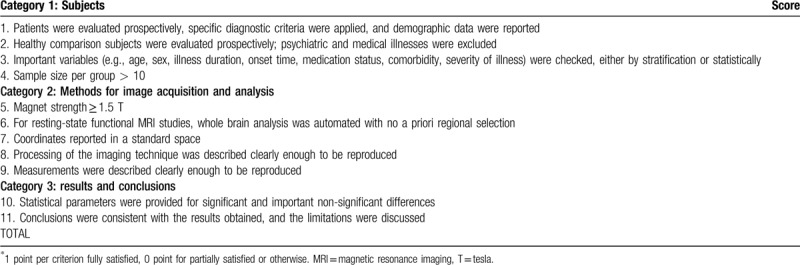
Quality assessment categories.

### 2.8 Data analysis

The collected data will be summarized in a table, including publication information, methodologies, and significant study findings. Methodologies and neuroimaging results then will be pooled and described in details. The total and average sample size, mean age of participants, and year of disease duration will be calculated, disease-related scales, QoL scales, and emotional scales will be summarized. A qualitative review will be performed to synthesize the structure and function brain alterations and correlations between these alterations and the clinical characteristics of patients with TTH. For more clear presentation, these findings will be integrated separately according to different neuroimaging modality. Since the AES-SDM has a higher sensitivity, overlap, and a good control of false positives,^[28]^ it will be launched to quantitatively synthesize the differences in cerebral structure and function between TTH patients and HC. The strength of evidence of this review will be evaluated using the checklist described above.

The brain regions with structure or function alterations will be voxel-wise meta-analyzed using the AES-SDM, which has been widely used in many other studies, such as Alzheimer disease, autism spectrum, schizophrenia, depression and bipolar disorder.^[39–42]^ The meta-analysis of TTH will be implemented with a standard inverse-variance weighted, random effects model. The main threshold will be set at an uncorrected *P* = .005, with a cluster extent of 10 voxels and SDM-Z > 1, as this threshold was found to be optimally balance sensitivity and specificity and adequately controlled the probability of detecting an effect by chance.^[28,43]^ Heterogeneity Q statistic maps (Chi^2^ distribution converted to z values) can be used to explore those brain regions with higher heterogeneity. Potential publication bias will be evaluated by using funnel plots and Egger test if the numbers of main findings are no less than 10 (main findings of structure and function separately).

#### 2.8.1 Multimodal analysis of structural and functional response

In order to locate the regions of brain structure and function alteration in TTH patients, the structural and functional findings will be cross-validated in a single map by computing the union of the structural and functional *P*-values.^[28,44]^

#### 2.8.2 Meta-regression or subgroup analysis

If sufficient studies are included, we will explore the following potential sources of heterogeneity using subgroup analyses (such as the different neuroimaging techniques of fMRI/VBM/ positron emission tomography, the 1.5/3.0 tesla scanner, and the FWHM) or meta-regression (such as the sample size, the demographic characters, the mean frequency, intensity, and duration of headache).

#### 2.8.3 Sensitivity analysis

A systematic whole-brain voxel-based jackknife sensitivity analysis will be conducted to test the replicability of the results, which consists of repeating the main analysis by systematically discarding each study and repeating the analysis, and to determine whether the previously significant brain regions remain significant.

## 3 Conclusion

Although neuroimaging has advantages in helping us to realize the pathophysiology of TTH in vivo, especially at the level of supraspinal structures, not all studies of the brain structure and function alterations in TTH have completely reported consistent findings, nor have summarized related evidence. Therefore, this SR and meta-analysis will be launched, aiming to improve the understanding of the pathophysiology of TTH, as well as propose avenues for future neuroimaging studies of TTH.

## Author contributions

JZ, SRC, HY conceived the review protocol and drafted the manuscript. MLL and ZJL revised the study design. JZ, HY, ZHY and LL participated in the design of the search strategy and data extraction data set. SRC, XGX and YJC formed the data synthesis and analysis plan. In practice, LZJ and MLL will monitor each procedure of the review and are responsible for the quality control. All authors reviewed the manuscript.
